# Osteopathy in Congress

**Published:** 1907-02

**Authors:** 


### OSTEOPATHY IN CONGRESS.

---

At the last session of Congress the Senate passed a bill (S. 5521) to regulate the practice of osteopathy, to license osteopathic physicians and to punish persons violating the provisions in the District of Columbia. The measure calls for a board "to be composed of five physicians in good standing—adherents of the osteopathic system of practice." This board is to be appointed by the commissioners of the district and should have the usual number of officers.

The bill provides: "From and after the passage of this act all persons desiring to practice osteopathy in the District of Columbia shall apply to said board for a license to do so. Applicants shall submit to examination on the following named branches, to wit: anatomy, physiology, chemistry, pathology, principles and practice of osteopathy, hygiene, histology, surgery, obstetrics and gynecology, medical jurisprudence and such other branches as said board shall deem advisable; but said board shall not examine any applicant until satisfactory proof is furnished that he or she is of good moral character and over twenty-one years of age, nor until he or she has presented a diploma issued to him or her by a reputable college of osteopathy, which, at the time said diploma was issued, required personal attendance on a course of instruction of not less than twenty-seven months. All examinations shall be both theoretical and practical and of sufficient severity to test the candidate's fitness to practice osteopathy. All questions propounded for the examinations of applicants, except such as relate specifically to the treatment of disease, shall be the same as questions to applicants for licenses to practice medicine under the provisions of an act entitled 'An act to regulate the practice of medicine and surgery, to license physicians and surgeons, and to punish persons violating the provisions thereof in the District of Columbia,' approved June 3, 1896, and the answers submitted by applicants for licenses to practice osteopathy shall be marked on a scale of severity equivalent to that adopted for the marking of the answers submitted by applicants for licenses to practice medicine. To the end of aforesaid the president of the board of osteopathic examiners, in so far as relates to the selection of questions to be used in examinations and in so far as relates to the rating of the answers submitted by applicants for licenses to practice osteopathy or for licenses to practice medicine, shall be entitled to all the rights and privileges of the presidents of the several boards of medical examiners of said district."

This bill is remarkable in several particulars: In the first place it is a recession from the position declared by the promoters of this cult that osteopathy per se is a sufficient remedy with which to successfully treat all the ills to which flesh is heir. It now becomes apparent that these practitioners are to be licensed in surgery, obstetrics, gynecology, which, according to the phraseology of the measure, are separate and distinct from the principles and practice of osteopathy. This contradiction does away with the subterfuge under which osteopathy gained a footing in the various states, namely, that they were not physicians within the meaning of the law; that they did not treat disease in such a way or by such metdods as to bring them properly within the purview of the law. This forfeiture of their claims is not, however, associated with a forfeiture of their demands for special privileges. The special privilege asked for and granted, so far as the U. S. Senate is concerned, to the representatives of this cult in Washington, consists of an evasion of an examination of fundamental branches under conditions imposed on all other applicants to practice the healing art. The provision that the questions shall be identical and that the grading shall be done on a scale of "equivalent severity" has again made surrender of the entire contention so far as the osteopaths are concerned. For, if the questions are identical and the grading is to be done on a scale of "equivalent severity," why should there be another set of persons designated to ask questions and to do the grading?

The whole thing, considered as judiciously as it may be, can only be construed as an effort on the part of persons deficient in fundamental qualifications to acquire the right to treat disease and thus assume responsibilities for which they are not qualified.

But the most pernicious feature of the whole measure is the effort on the part of the promoters of this measure to maintain and perpetuate the spirit of sectarianism in the profession in which a fundamental science is to be recognized as a unity. The advanced steps taken by the American Medical Association have gone far toward putting an end to this sort of thing by obliterating, in large measure, the lines of demarkation that define sectariamism in medicine. The promoters of this measure have sought to negative this, so far as the District of Columbia is concerned and so far as the influence of the bill passed by the National Congress is calculated to establish a precedent.—*Ed. Jour. A. M. A , Jan. 5th, '07.*

